# Visual Working Memory Recruits Two Functionally Distinct Alpha Rhythms in Posterior Cortex

**DOI:** 10.1523/ENEURO.0159-22.2022

**Published:** 2022-10-04

**Authors:** Julio Rodriguez-Larios, Alma ElShafei, Melanie Wiehe, Saskia Haegens

**Affiliations:** 1 Department of Psychiatry, Columbia University, New York, NY, 10032; 2 Division of Systems Neuroscience, New York State Psychiatric Institute, New York, NY, 10032; 3 Donders Institute for Brain, Cognition and Behavior, Radboud University, Nijmegen, 6525 EN, The Netherlands

**Keywords:** alpha oscillations, attention, MEG, memory

## Abstract

Oscillatory activity in the human brain is dominated by posterior alpha oscillations (8–14 Hz), which have been shown to be functionally relevant in a wide variety of cognitive tasks. Although posterior alpha oscillations are commonly considered a single oscillator anchored at an individual alpha frequency (∼10 Hz), previous work suggests that individual alpha frequency reflects a spatial mixture of different brain rhythms. In this study, we assess whether independent component analysis (ICA) can disentangle functionally distinct posterior alpha rhythms in the context of visual short-term memory retention. Magnetoencephalography (MEG) was recorded in 33 subjects while performing a visual working memory task. Group analysis at sensor level suggested the existence of a single posterior alpha oscillator that increases in power and decreases in frequency during memory retention. Conversely, single-subject analysis of independent components revealed the existence of two dissociable alpha rhythms: one that increases in power during memory retention (Alpha1) and another one that decreases in power (Alpha2). Alpha1 and Alpha2 rhythms were differentially modulated by the presence of visual distractors (Alpha1 increased in power while Alpha2 decreased) and had an opposite relationship with accuracy (positive for Alpha1 and negative for Alpha2). In addition, Alpha1 rhythms showed a lower peak frequency, a narrower peak width, a greater relative peak amplitude and a more central source than Alpha2 rhythms. Together, our results demonstrate that modulations in posterior alpha oscillations during short-term memory retention reflect the dynamics of at least two distinct brain rhythms with different functions and spatiospectral characteristics.

## Significance Statement

alpha Oscillations are the most prominent feature of the human brain’s electrical activity, and consist of rhythmic neuronal activity in posterior parts of the cortex. alpha Is usually considered a single brain rhythm that changes in power and frequency to support cognitive operations. We here show that posterior alpha entails at least two dissociable rhythms with distinct functions and characteristics. These findings could solve previous inconsistencies in the literature regarding the direction of task-related alpha power/frequency modulations and their relation to cognitive performance. In addition, the existence of two distinct posterior alpha rhythms could have important consequences for the design of neurostimulation protocols aimed at modulating alpha oscillations and subsequently cognition.

## Introduction

Working memory entails the storage of information over brief periods of time for its later manipulation (Repovs and Baddeley, 2006; Baddeley, 2010). Traditionally, working memory has been linked to the prefrontal cortex, as neurons in this area show increased spiking when information has to be transiently stored (Fuster and Alexander, 1971; D’Esposito and Postle, 2015). However, more recent research has shown that the brain mechanisms supporting working memory are not limited to the activity of individual neurons in a specific part of the cortex (Miller et al., 2018). Rather, memory traces are distributed in the brain, involving areas beyond the prefrontal cortex (Christophel et al., 2017, 2018). Moreover, the transient maintenance of information involves changes at network level that cannot be well addressed when studying the activity of single neurons (Miller et al., 2018). Neural oscillations, which reflect the summed activity of neural populations (Cohen, 2017), are thought to play an important role in the transient storage of information in the brain (Lundqvist et al., 2016, 2018; Wolinski et al., 2018).

In the human brain, neural oscillations are dominated by alpha rhythms (8–14 Hz; Hari et al., 1997; Bazanova and Vernon, 2014). Although alpha rhythms are most prominent in posterior areas, they are also found in sensorimotor and auditory cortex (commonly referred to as “mu” and “tau” rhythms, respectively; Lehtelä et al., 1997; Pfurtscheller et al., 2000; Haegens et al., 2011; Bastarrika-Iriarte and Caballero-Gaudes, 2019). Previous work has shown that alpha oscillations desynchronize (decrease in power) in task-relevant areas and synchronize (increase in power) in task-irrelevant areas in a wide variety of cognitive tasks (Klimesch, 1999; Jensen et al., 2002; Jokisch and Jensen, 2007; Haegens et al., 2009). Based on these results, it has been proposed that alpha’s function is to gate information through the brain via functional inhibition (Klimesch et al., 2007; Jensen and Mazaheri, 2010). In line with this idea, recent research in humans demonstrates that the amplitude of alpha oscillations is negatively associated with neural excitability (Chapeton et al., 2019; Haegens et al., 2022; Iemi et al., 2022).

Posterior alpha oscillations are thought to be especially relevant in visual working memory (de Vries et al., 2020). Although several studies have shown significant power modulations of posterior alpha rhythms when visual information has to be transiently stored, the direction of these power modulations is highly inconsistent (Pavlov and Kotchoubey, 2022). Studies reporting increased alpha power during visual memory maintenance argue that this increase is aimed to block visual input by inhibiting (task-irrelevant) occipital and/or parietal areas (Jensen et al., 2002; Tuladhar et al., 2007; Bonnefond and Jensen, 2012). In contrast, studies reporting posterior alpha power decreases during memory retention argue that these occipitoparietal areas are actually task-relevant and need to be disinhibited to support the short-term storage of visual information (van Ede et al., 2017; de Vries et al., 2018; Erickson et al., 2019).

A possible explanation for the inconsistent findings regarding posterior alpha modulations during visual working memory is the existence of multiple alpha rhythms. More than 20 years ago, Klimesch and colleagues proposed a division of the alpha band in different sub-bands (i.e., “upper alpha” and “lower alpha”) based on their differential power modulations during a wide range of cognitive tasks (Klimesch et al., 1993; Klimesch, 1999). However, since alpha rhythms were not spatially separated in these studies, it was not possible to assess whether power modulations in the two alpha sub-bands reflected the activity of two different oscillators or a change in frequency of a single oscillator (Haegens et al., 2014; Mierau et al., 2017). Later studies that did perform source separation supported the idea of multiple posterior alpha rhythms. Specifically, spatially distinct posterior alpha rhythms have been identified in the context of resting state (Barzegaran et al., 2017), visual perception (Gulbinaite et al., 2017; Benwell et al., 2019) and multimodal attention (Sokoliuk et al., 2019).

Previous literature has shown that independent component analysis (ICA) is a powerful analytical tool to separate brain rhythms in Magneto- and Electro-Encephalography (M/EEG) (Debener et al., 2005; Wagner et al., 2018; Benwell et al., 2019). ICA is a blind source separation technique that aims to identify signals whose time courses convey maximally distinct information. When applied to M/EEG data, ICA is thought to be able to separate locally synchronous activity from distinct “cortical patches” (i.e., compact neuronal populations with strong short-range anatomic connections; Onton et al., 2006). The main advantage of ICA relative to other source localization techniques is that it simplifies the M/EEG inverse problem by modeling which signals are contained in the volume conducted scalp data instead of estimating the projection weights of all possible sources (Delorme et al., 2012).

In this study, we use ICA to examine whether alpha power modulations in posterior regions during short-term memory retention reflect the activity of one or several brain rhythms. We acquired magnetoencephalography (MEG) during a visual working-memory task in which participants (*N* = 33) had to briefly remember one out of four spatial directions, while task difficulty was modulated by introducing visual distractors. We first assessed alpha dynamics at sensor level by comparing alpha power and frequency between fixation and memory retention. Then, we performed the same comparison using statistically independent posterior sources as extracted through ICA (Delorme et al., 2012). Interestingly, while results at sensor level suggested a change in power and frequency of a single posterior alpha rhythm during memory retention, our ICA results demonstrated that posterior alpha dynamics reflect the activity of at least two alpha rhythms with distinct functions and spatiospectral characteristics. We believe that these results have important implications not only for the analysis and interpretation of alpha oscillations in M/EEG but also for their potential modulation through different neurostimulation techniques.

## Materials and Methods

### Participants

A total of 35 healthy right-handed adult participants (mean age 25.2 years, range 20–33; 17 female, 18 male) took part in the experiment. Participants reported normal or corrected-to-normal vision and no history of neurologic or mental illnesses. Before the experiment, participants were informed about the MEG system as well as safety regulations and signed an informed consent form. The study falls under the general ethics approval (CMO 2014/288 “Imaging Human Cognition”) in accordance with the Declaration of Helsinki. After participation, the participants received a monetary reward. Two participants were excluded because of technical problems during data acquisition.

### Experimental design

**Figure 1.**
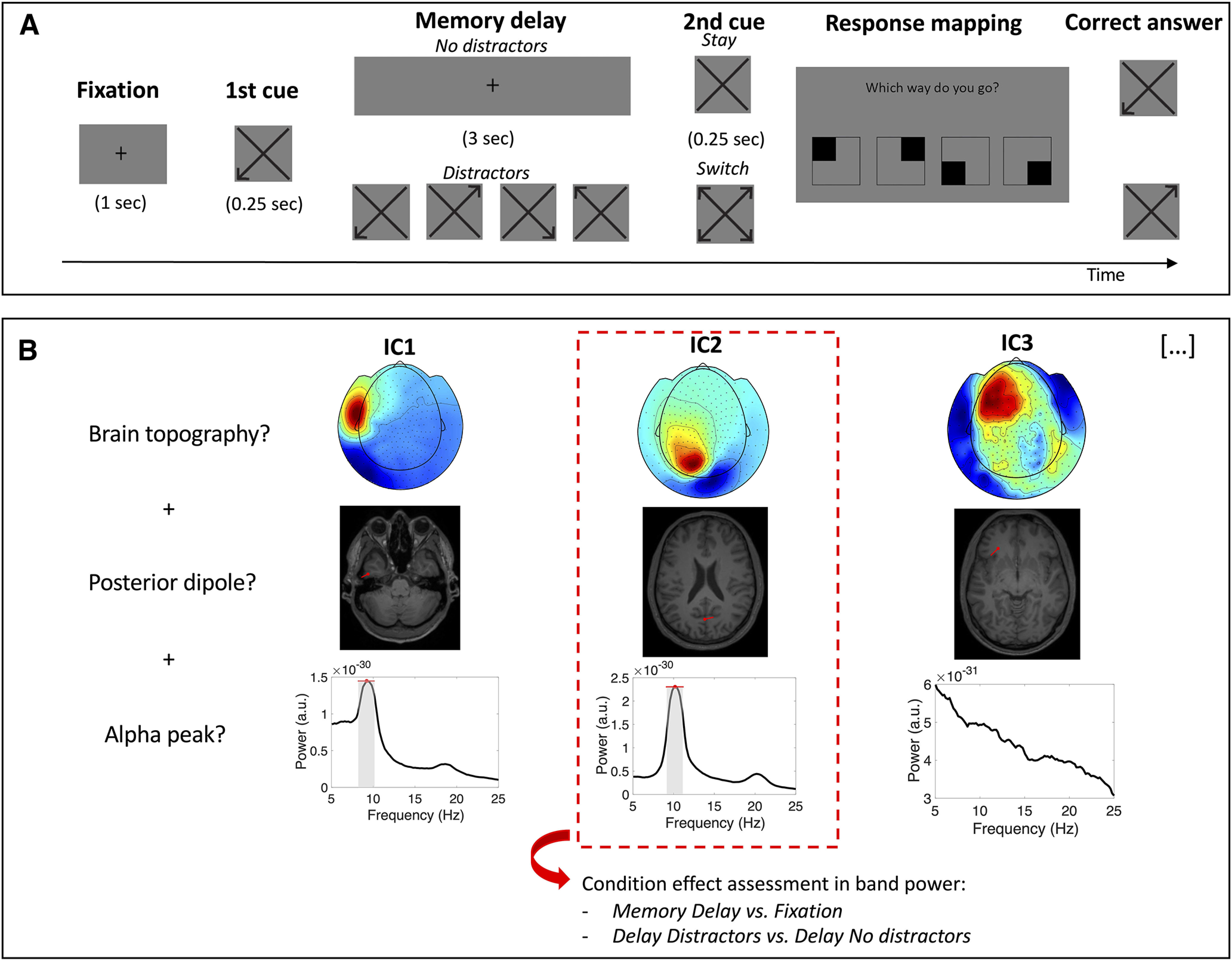
Task and main analytical approach. ***A***, Task schematic. Participants were asked to remember a visual direction cue (i.e., upper left, upper right, bottom left, or bottom right) for 3 s (memory delay). In 50% of the trials, the delay period contained four visual distractors. Based on the content of a second cue (i.e., “stay” or “switch”), participants had to report the direction of the first cue or its opposite. ***B***, ICA-based selection in an exemplary subject. A selection of independent components was performed based on their topography (top panel), estimated source (middle), and spectrum (bottom). Only independent components with a brain topography (IClabel algorithm classification > 0.80), an estimated dipole in occipital or parietal cortex and a peak in the α range were selected for analysis (in this example, the component marked in the red rectangle). The frequency band of each independent component was defined based on the average spectrum (peak frequency and width; gray area). Condition-related modulations in band power (i.e., delay vs fixation, distractor vs no distractor) were assessed through single-trial analysis of each independent component.

MEG was recorded while participants performed a visual working memory task (Fig. 1*A*). This task was designed to emulate real-life situations in which participants would navigate in a city using directions that they had to keep in mind for a short period of time. The goal of the task was to remember one out of four directions. Participants were first presented with a visual direction cue (0.25 s) pointing to the upper left, upper right, bottom left, or bottom right. After a delay period (3 s), a second cue was presented (0.25 s). The second cue was either a “stay” cue, meaning that the correct answer was the direction indicated by the first cue, or a “switch” cue, indicating that the correct answer was the direction opposite to the first cue. After the second cue, a response mapping diagram was shown, indicating which button corresponded to which direction. The correspondence between each button and direction was randomized between blocks (a total of eight different response mapping diagrams were shown). Participants were instructed to answer as quickly and as accurately as possible, with the right hand via a button press. In 50% of the trials, the delay period contained four distractors (randomly drawn direction cues) that were presented at a 0.33-s interstimulus interval. Participants were instructed to ignore the distractors and to keep only the first cue in mind. Participants performed eight blocks of 48 trials (∼5 min each), with short breaks between blocks. Participants were seated upright in the MEG helmet and were instructed to keep their head position as stable as possible for the duration of the experiment. Before the MEG recording, participants performed a training block (16 trials) to make sure that they understood the task correctly. The experimental stimuli were programmed and presented with the software Presentation (version 18.0, Neurobehavioural Systems).

### Data acquisition

MEG data were recorded with a 275-channel CTF MEG system with axial gradiometers at a sampling rate of 1200 Hz (CTF MEG systems, VSM MedTech Ltd.). Six channels were disabled because of permanent malfunction. In order to monitor the head position of the participants and to allow for adjustments to the original position in between blocks, the real-time representation of the participant’s head position was monitored using three head localization coils at the right and left ear canals as well as the nasion (Stolk et al., 2013). These points were further used as offline anatomic landmarks to align the MEG data with structural images of the participant’s brain for source reconstruction. Further, movement of the left eye was tracked during the experiment using an Eyelink eyetracker (SR Research Ltd.). After the experiment, the participant’s head shape was digitized using a Polhemus 3D tracking device (Polhemus). In a separate session, an anatomic MRI scan of the participant’s brain was recorded, unless the participant’s scan could be obtained from the database of the institute. The MRI data were recorded with the 3T Siemens Magnetom Skyra MR scanner.

### MEG analysis

MEG analysis was performed using the Fieldtrip toolbox (Oostenveld et al., 2011), EEGlab (Delorme and Makeig, 2004), and custom MATLAB scripts (version R2021a).

#### Preprocessing

The raw continuous data were downsampled to 300 Hz and epoched relative to the first cue (from −1.5 to +10 s). A band-stop filter was applied at 50, 100, and 150 Hz to remove line noise and its harmonics. The data were visually inspected to reject trials with artifacts (e.g., muscle artifacts, SQUID sensor jumps). Next, the data were bandpass filtered between 3 and 30 Hz (Butterworth IIR filter) and ICA was computed (i.e., EEGlab implementation of the infomax ICA algorithm by Bell and Sejnowski, 1995). Finally, the IClabel algorithm (Pion-Tonachini et al., 2019) was used to classify components in the categories Brain, Muscle, Eye, Heart, Line Noise, Channel Noise, and Other based on their spatial topography.

#### Sensor-level analysis

First, independent components that were classified as muscle, eyes, heart or channel noise by the IClabel algorithm with a probability higher than 80% were discarded. Further analysis was performed at sensor level after back-projecting the remaining components. We computed the planar representation of the MEG field distribution from the single-trial data using the nearest-neighbor method. This transformation facilitates the interpretation of the sensor level data as it makes the signal amplitude maximal above its source (as implemented in Fieldtrip functions *ft_meg_planar* and *ft_combine_planar*). The power spectrum of each channel between 5 and 25 Hz was obtained using a multitaper frequency transformation (*ft_freqanalysis*). This transformation was done separately for the fixation (1 s) and the memory delay period (1-s window centered in the delay period). The data were zero-padded to 5 s to obtain a frequency resolution of 0.2 Hz. alpha Band power was estimated as the mean power values between 8 and 14 Hz across trials. Individual alpha power and frequency were estimated using the MATLAB *findpeaks* algorithm (i.e., maximum peak between 8 and 14 Hz).

#### Component-level analysis

A series of conditions were imposed to select posterior oscillatory components in the alpha range. First, independent components had to be classified as brain components by the IClabel algorithm with a probability higher than 80%. Second, independent components had to show a maximum peak in the alpha range (as detected with the MATLAB *findpeaks* function). Third, independent components needed to project to a single dipole in occipital or parietal cortex. For that purpose, the whole brain was scanned with a single dipole to find the location where the dipole model was best able to explain the topography of each independent component (*ft_dipolefitting*). The source localization of dipoles was done using individual T1-weighted anatomic images of the participants’ brains. For that, individual MRIs were first normalized in MNI space (*ft_volumenormalise*) and segmented (*ft_volumesegment*). Then a realistic single-shell headmodel was computed (*ft_prepare_headmodel)* based on the surface mesh obtained from the segmented MRI (*ft_prepare_mesh*; Nolte, 2003). Finally, to automatically identify dipoles that were located in occipital and parietal cortices we used the AAL atlas (Tzourio-Mazoyer et al., 2002) available in the Fieldtrip toolbox (Oostenveld et al., 2011). The frequency transformation of independent components was identical to the one used for sensor level analysis. The only difference is that to estimate alpha power per condition in single trials, the individual alpha band was previously defined per component based on its average power spectrum (across trials; Fig. 1*B*). This approach was adopted to compensate for the lower signal-to-noise ratio of oscillatory activity when estimated in single trials. For visualization purposes, independent components were also transformed in the time-frequency domain (5–25 Hz) using Morlet wavelets of six cycles (function *BOSC_tf*; see Whitten et al., 2011).

A similar approach was adopted to estimate peak frequency in single trials. In this case, independent components were first filtered (MATLAB function *firl1*) around their individual alpha band, after which instantaneous frequency was estimated with the method developed in Cohen (2014). In short, instantaneous frequency was computed by multiplying the first temporal derivative of the phase angle time series (extracted through its Hilbert transform) by the sampling rate and dividing it by 2π. Then, a median filter was applied to the instantaneous frequency time series (10 steps between 10 and 400 ms) to attenuate nonphysiological frequency jumps. Instantaneous frequency was averaged within the fixation period and the memory delay (1-s windows) to get an estimation of peak frequency in each period.

### Statistical analysis

#### Behavioral data

Repeated-measures ANOVA was performed with the JASP software (Love et al., 2019), *post hoc* paired samples *t* test were performed in MATLAB R2021a. The effect size was estimated as eta squared (η^2^) for the ANOVA and as Cohen’s *d* for the *t* tests.

#### MEG data

The comparison of MEG parameters of interest between conditions was performed using paired samples and independent samples *t* tests (MATLAB R2021a implementation). For the comparison of the parameters of interest in independent components at single-trial level (i.e., power/frequency during memory retentions vs delay), we employed Wilcoxon signed-rank test (MATLAB R2021a) to minimize the effect of possible outliers because they are more likely to occur when analyzing single trials (Cohen and Cavanagh, 2011). In order to assess a possible relationship between MEG parameters and accuracy, a median split approach was adopted. In short, trials were divided into two groups based on the median of the MEG parameter (i.e., high and low alpha power) and mean accuracy was compared between “high” and “low” trials at group level (paired-samples *t* tests). We corrected for multiple comparisons using the false discovery rate (FDR) method (Benjamini and Hochberg, 1995). Effect size was estimated through Cohen’s *d* estimate.

### Data and code accessibility

MEG data and MATLAB scripts to reproduce our findings will be made publicly available through the Open Science Framework (OSF) webpage: https://osf.io/h56b3/.

## Results

### Behavioral performance

Mean accuracy was 87.5% across conditions (SD = 19.4). Repeated-measures ANOVA revealed a significant main effect of the factor distractor (*F*_(1,32)_ = 11.54; *p* = 0.002; η^2^ = 0.102). *Post hoc* paired sample *t* tests showed that accuracy was greater for the no distractor than for the distractor condition (*t*_(32)_ = −3.39; *p* = 0.0018; *d* = 0.59). No main effect of rule (i.e., stay vs switch) or rule by distractor interaction was found.

Mean reaction time was 698 ms across conditions (SD = 255). Repeated-measures ANOVA revealed a main effect of rule (*F*_(1,32)_ = 8.79; *p* = 0.006; η^2^ = 0.12). *Post hoc* paired sample *t* tests showed that reaction time was shorter for stay versus switch rules (*t*_(32)_ = 2.96; *p* = 0.0057; *d* = 0.51). No effect of distractor nor rule by distractor interaction was found. Note that since the decision-button association changed in each trial, reaction times are difficult to interpret in this task. For this reason, we focused on accuracy to assess brain-behavior associations.

### Posterior alpha peak at sensor-level increases in power and decreases in frequency during memory retention

**Figure 2.**
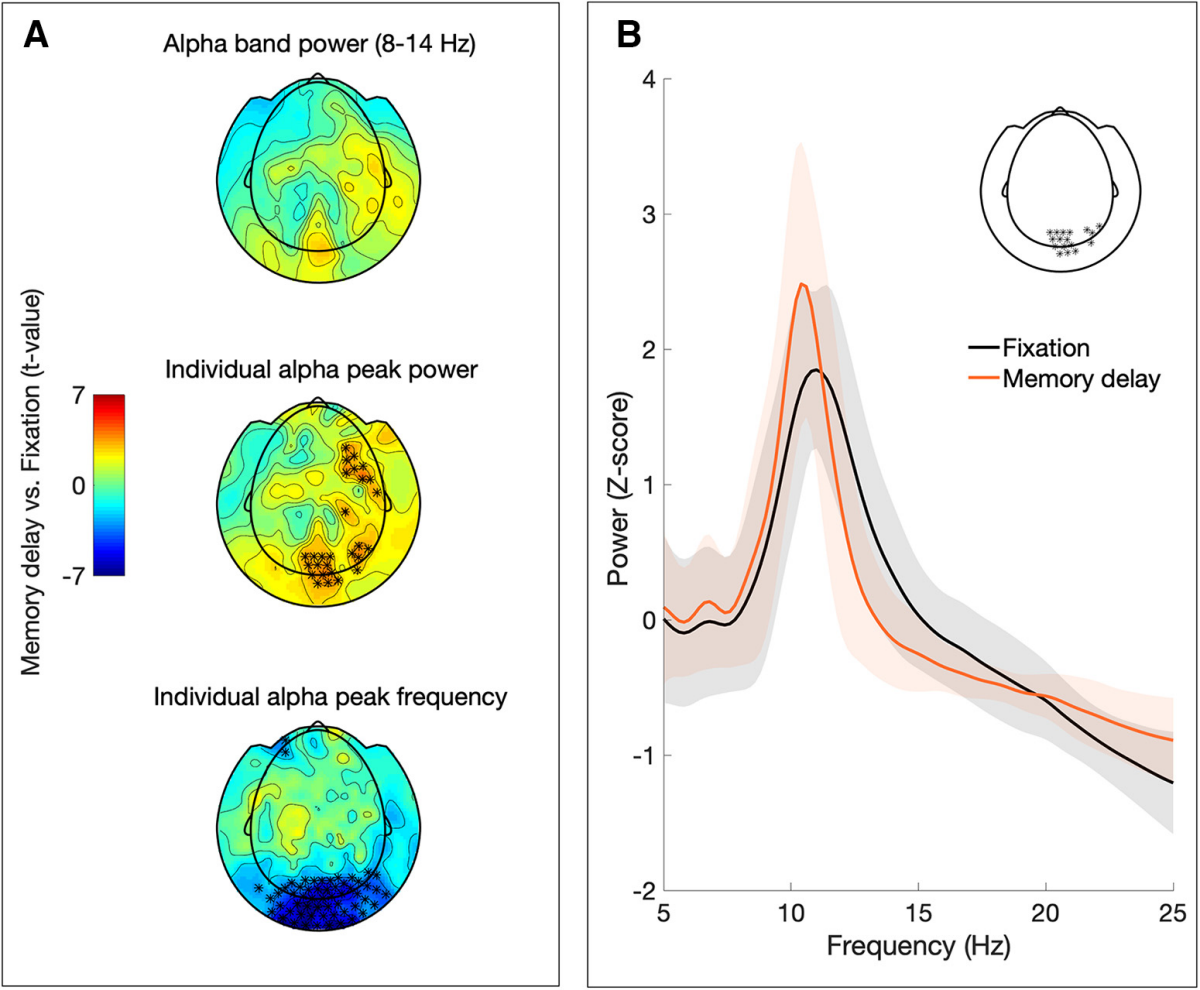
Sensor level analysis. ***A***, Topographic plots depicting the *t* values from the condition comparison (memory delay vs fixation) in α band power (top), individual α peak power (middle), and individual α peak frequency (bottom). Significant differences (*p* < 0.05 after FDR correction) are marked with asterisks. ***B***, Mean power spectrum for fixation (black graph) and memory delay (orange) of sensors showing significant changes in individual α peak power and frequency (shaded area depicts SD across subjects; sensors included in spectra indicated in inset).

No significant differences between conditions in alpha power were found when estimated using an a priori definition of the alpha band (8–14 Hz; Fig. 2*A*, top plot). However, individual alpha peak power showed a significant increase during the memory delay in posterior and right frontocentral sensors (*p* < 0.05 after FDR correction; Fig. 2*A*, middle plot). In addition, individual alpha frequency decreased significantly in posterior and frontal sensors (*p* < 0.05 after FDR correction; Fig. 2*A*, bottom plot). Hence, some posterior sensors showed a significant modulation in both individual alpha peak power and frequency (Fig. 2*B*). No significant distractor effect (comparison of distractor vs no distractor conditions) or relation to accuracy (median split approach) were found in either individual alpha power or frequency at sensor level.

### ICA reveals two distinct alpha components based on their power modulations during the memory delay

**Figure 3.**
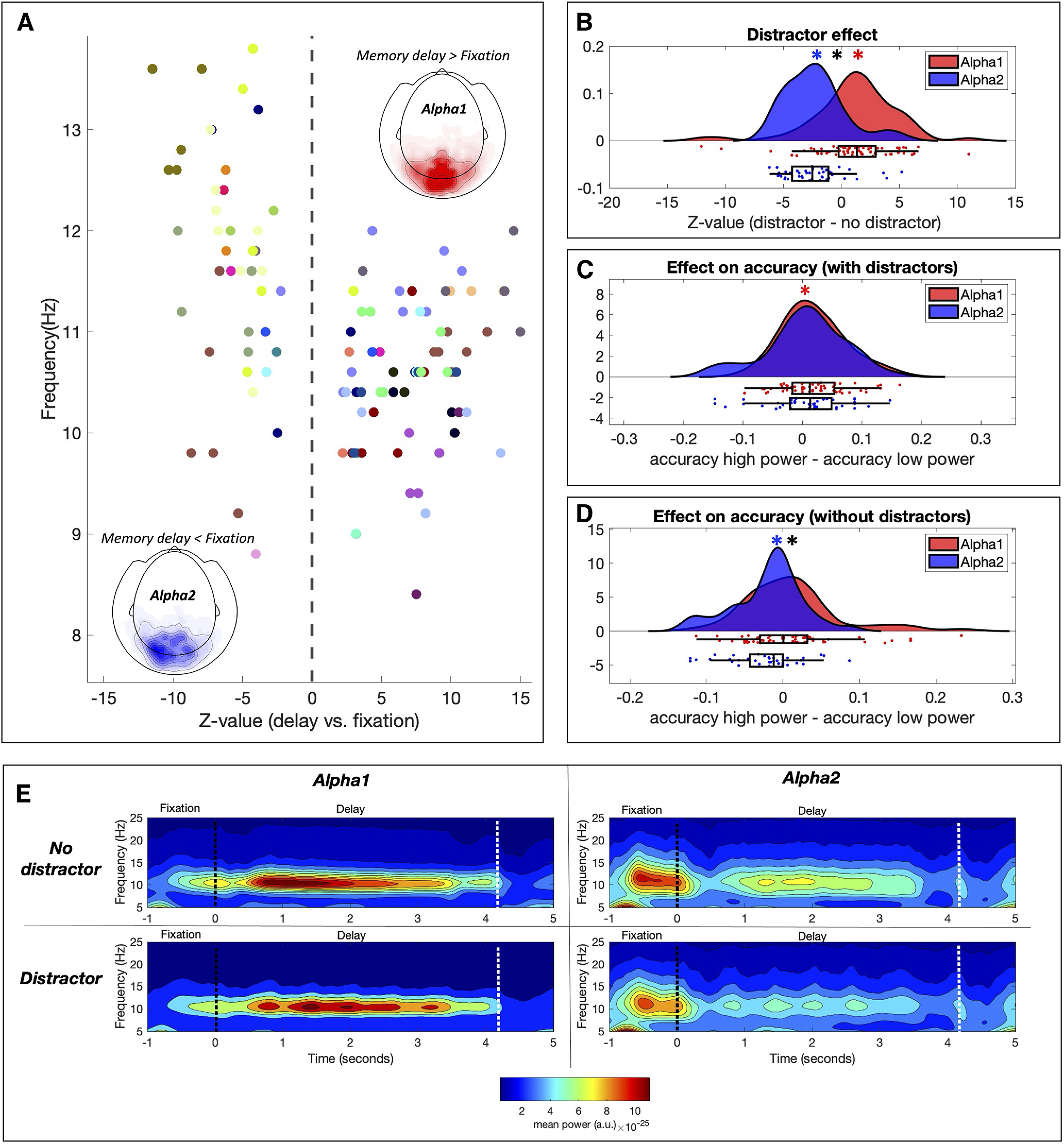
Power modulations of independent α components. ***A***, Power changes during the memory delay relative to fixation. Z-values (representing the memory retention effect in each component) are plotted as a function of their peak frequency (each color codes for a different subject). Components showing a significant increase in power during the memory delay were denominated Alpha1 while components showing a significant decrease were denominated Alpha2. The mean topography of the power change is plotted separately for Alpha1 (red) and Alpha2 (blue) components. ***B***, Differential distractor-related power modulations of Alpha1 and Alpha2 components. Z-values represent the distractor effect in individual components. At group level, Alpha1 components (red) showed significantly more power in the presence of distractors while Alpha2 components (blue) showed significantly less. Colored asterisks mark statistical significance (*p* < 0.05) of the Alpha1 (red) or Alpha2 (blue) distributions against 0 (one-sample *t* test). Black asterisks mark significant differences (*p* < 0.05) between Alpha1 and Alpha2 distributions. ***C***, Effect of power modulations on accuracy in the distractor condition. Accuracy was compared for high power and low power trials (median split), showing a significantly greater accuracy for trials with high Alpha1 power in the distractor condition. ***D***, Same as ***C*** for the no-distractor condition, showing a significantly lower accuracy for trials with high Alpha2 power in the no-distractor condition. ***E***, Average time-frequency representation of Alpha1 and Alpha2 components for the distractor and the no distractor conditions. The black vertical line indicates the presentation of the first cue. The white vertical line marks the presentation of second cue.

In order to assess whether the reported changes in posterior alpha power reflect the activity of one or several brain rhythms, we performed the same condition comparisons that we performed at sensor level using independent posterior alpha components (Fig. 1*B*). We found a total of 170 posterior alpha components (across subjects) based on their topography at sensor level, their spectrum, and their estimated source. The power of 111 components was significantly modulated during the memory delay relative to fixation (*p* < 0.05 after FDR correction; mean number of components per subject = 3.3; SD = 2.9). Unlike sensor-level analysis, the comparison of posterior alpha components in single subjects revealed both increases and decreases in alpha power during the memory delay. Specifically, the power of 68 alpha components showed a significant increase during memory delay relative to fixation (hereafter called Alpha1), while the power of 43 alpha components showed a significant decrease (hereafter called Alpha2; Fig. 3*A*). The breakdown across subjects was as follows: nine subjects showed both types of alpha components, 11 subjects showed Alpha1 components only, seven subjects showed Alpha2 components only, and six subjects showed no significant alpha components.

In addition, we assessed whether the frequencies of Alpha1 and Alpha2 components were differentially modulated during memory delay. However, we did not find significant differences between Alpha1 and Alpha2 components in frequency modulations associated with memory retention (*t*_(109)_ = −1.45; *p* = 0.14; *d* = 0.28). Both Alpha1 and Alpha2 components significantly decreased in frequency during the memory delay relative to fixation (*t*_(67)_ = −8.18, *p* < 0.001; *d* = 1.00; *t*_(42)_ = −6.98, *p* < 0.001; *d* = 1.07).

In summary, single-subject analysis of posterior alpha components demonstrates the existence of at least two distinct rhythms (Alpha1 and Alpha2) based on their opposite power modulations during memory retention.

### Alpha1 and Alpha2 show opposite distractor-related power modulations

In order to assess whether the power of Alpha1 and Alpha2 components was differentially modulated in the presence of distractors, we first estimated the distractor effect in individual components (distractor vs no distractor) through Wilcoxon signed rank tests. Then we tested at group level whether the z-values of Alpha1 and Alpha2 components differed significantly from 0 (one sample *t* test) and from each other (independent samples *t* test). We found that alpha components that increased in power during the memory delay (i.e., Alpha1) showed a significant power increase in the presence of distractors (*t*_(67)_ = 2.66; *p* = 0.0097; *d* = 0.32) while alpha components that decreased in power during the memory delay (i.e., Alpha2) showed a significant power decrease in the presence of distractors (*t*_(42)_ = −5.78; *p* < 0.001; *d* = 0.88). Hence, Alpha1 and Alpha2 showed opposite and significantly different (*t*_(109)_ = 5.42; *p* < 0.001; *d* = 1.05) distractor-related power modulations (Fig. 3*B*,*E*).

### Alpha1 and Alpha2 power modulations have an opposite relation to accuracy

In order to assess the behavioral relevance of Alpha1 and Alpha2 power modulations, we compared the accuracy between trials with high and low alpha power during the delay (% change from fixation). Since the presence of distractors was associated with lower accuracy, we performed this analysis for distractor and no-distractor conditions separately. For the distractor condition, accuracy was significantly higher for trials showing greater Alpha1 power (*t*_(67)_ = 2.97; *p* = 0.0041; *d* = 0.36), while no differences were found in Alpha2 power (*t*_(42)_ = 0.54; *p* = 0.58; *d* = 0.08). However, power-related differences in accuracy between Alpha1 and Alpha2 did not reach statistical significance (*t*_(109)_ = 1.18; *p* = 0.24; *d* = 0.23). For the no-distractor condition, accuracy was significantly higher for trials with lower Alpha2 power (*t*_(42)_ = −2.79; *p* = 0.0078; *d* = 0.42), while no significant difference was found in Alpha1 power (*t*_(67)_ = 1.42; *p* = 0.15; *d* = 0.17). In this case, power-related differences in accuracy between Alpha1 and Alpha2 components did reach statistical significance (*t*_(109)_ = 2.76; *p* = 0.0067; *d* = 0.53).

In summary, in the presence of visual distractors, better accuracy was associated with higher Alpha1 power, while in the absence of visual distractors, better accuracy was associated with lower Alpha2 power.

### Alpha1 and Alpha2 components differ in their spatiospectral characteristics

**Figure 4.**
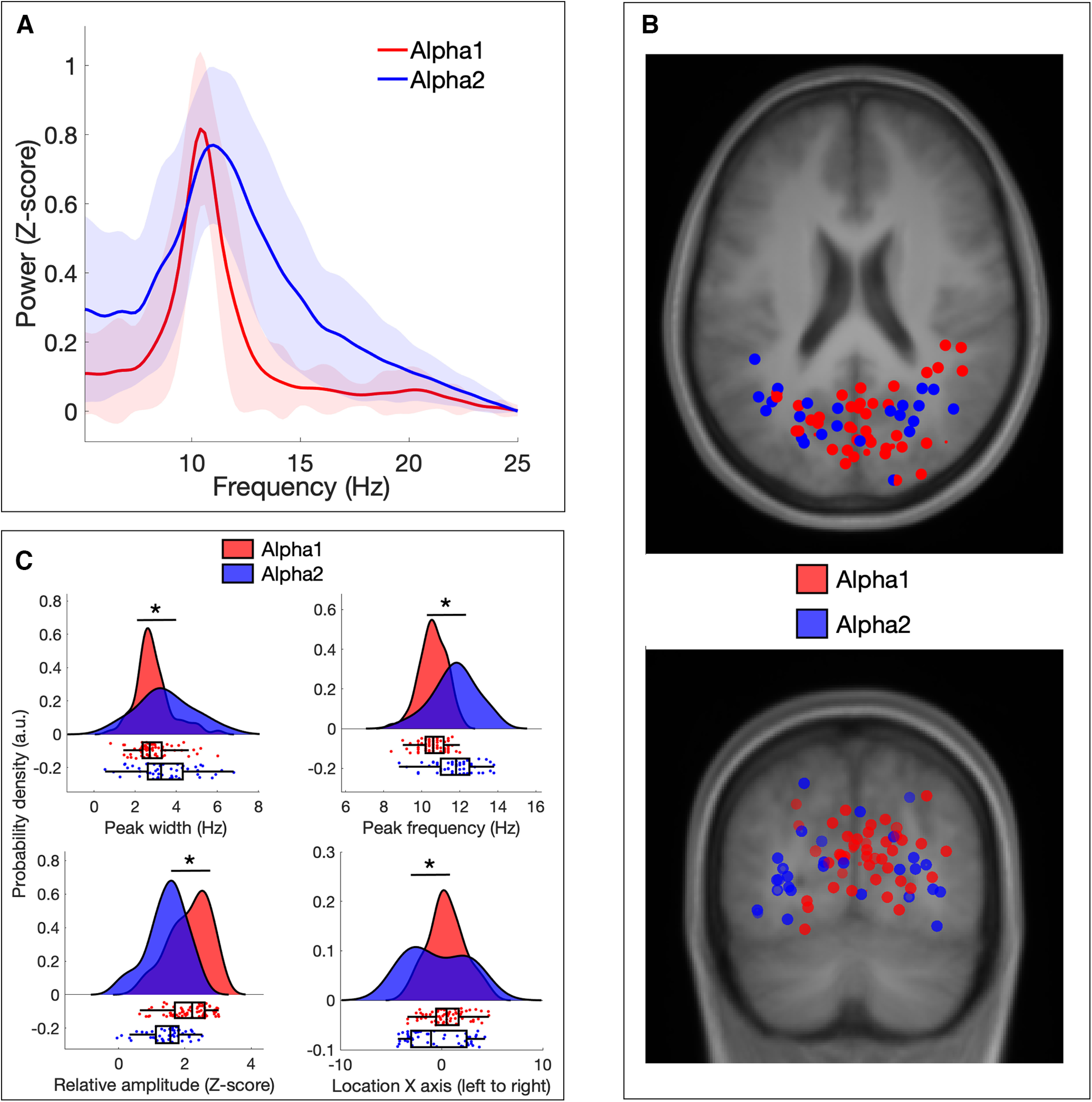
Spatiospectral differences between Alpha1 and Alpha2 components. ***A***, Average spectrum of Alpha1 and Alpha2 components across the complete trial (shaded area depicts SD). ***B***, Source localization of Alpha1 (red) and Alpha2 (blue) components as estimated through dipole fitting. The top panel shows the horizontal plane while the bottom panel shows the coronal plane. ***C***, Plots depicting significant differences in four spatiospectral parameters: peak width (top left panel), peak frequency (top right), relative amplitude (bottom left) and location (bottom right).

In order to assess whether Alpha1 and Alpha2 rhythms differ in their spatiospectral characteristics, we compared three different spectral parameters (peak frequency, peak width, and relative amplitude; Fig. 4*A*) and the location of their estimated main source through dipole fitting (*x*-, *y*-, and *z*-axes; Fig. 4*B*). This analysis revealed that Alpha1 components tended to have a lower peak frequency (*t*_(109)_ = −6.00; *p* < 0.001; *d* = 1.16), a narrower peak width (*t*_(109)_ = −2.43; *p* = 0.016; *d* = 0.47), a greater relative peak amplitude (*t*_(109)_ = 5.68; *p* < 0.001; *d* = 1.10), and a more central source estimation (*t*_(109)_ = 2.86; *p* = 0.005; *d* = 0.55) than Alpha2 components (Fig. 4*C*). No significant differences were found between the source estimation of Alpha1 and Alpha2 components in the other two axes (i.e., ventral to dorsal and posterior to anterior).

## Discussion

In this study we used ICA to examine whether posterior alpha power modulations during visual working memory reflect the dynamics of one or several brain rhythms. We recorded MEG while participants (*N* = 33) performed a visual working-memory task in which one out of four spatial directions had to be remembered for a short period of time. Task difficulty was modulated by introducing visual distractors during memory retention. Group analysis at sensor level suggested that posterior alpha consists of a single oscillator that increases in power and decreases in frequency during memory retention. In contrast, the analysis of independent components in single subjects revealed the existence of an alpha rhythm that increases in power during the memory delay (Alpha1), and an alpha rhythm that decreases in power during the memory delay (Alpha2). Interestingly, the power of Alpha1 and Alpha2 rhythms was differentially modulated by the presence of distractors (Alpha1 increased in power while Alpha2 decreased) and had an opposite relationship with accuracy (positive for Alpha1 and negative for Alpha2). In addition, Alpha1 and Alpha2 rhythms differed significantly in their spatiospectral characteristics. Specifically, Alpha1 rhythms showed a lower peak frequency, a narrower peak width, a greater relative peak amplitude and a more central source than Alpha2 rhythms. Thus, our results show that modulations in posterior alpha oscillations during memory retention reflect the dynamics of at least two distinct brain rhythms with different functions and spatiospectral characteristics.

The existence of alpha rhythms that increase and decrease in power during memory retention depending on their spatial origin is in line with prevailing theories of alpha function (Klimesch et al., 2007; Jensen and Mazaheri, 2010). According to these theories, alpha power reflects local cortical inhibition and therefore should increase in task-irrelevant areas and decrease in task-relevant areas. In the context of memory retention, it can be predicted that brain regions that are relevant to the transient maintenance of visual information would show alpha power decrease (disinhibition). On the other hand, brain regions that are irrelevant for memory maintenance (and could potentially interfere with the task) would show alpha power increase (inhibition). Although source estimation through dipole fitting in M/EEG has to be interpreted with caution (Leahy et al., 1998), the spatial distribution of the two observed types of alpha components suggests that, at least in occipital cortex, Alpha1 rhythms mostly originate in early visual areas while Alpha2 rhythms localize to higher-order areas (Fig. 4*B*). This is supported by their spectral profiles, since higher-order areas are thought to show a more pronounced 1/f trend (Ibarra Chaoul and Siegel, 2021) and higher peak frequency (Lundqvist et al., 2020), i.e., in line with what we see in Alpha2 components when compared with Alpha1 (Fig. 4*A*). Based on these results and previous evidence (Tuladhar et al., 2007; Popov et al., 2017; de Vries et al., 2018), we speculate that Alpha1 power increases reflect the inhibition of lower-order areas involved in visual processing, while Alpha2 power decreases reflect the disinhibition of higher-order areas supporting the transient storage of visual information.

If Alpha1 and Alpha2 rhythms during memory retention reflect the inhibition and disinhibition of task-irrelevant and task-relevant areas respectively, we would expect that behavioral performance improves when Alpha1 power increases and Alpha2 power decreases. Interestingly, Alpha1 power increases and Alpha2 power decreases were associated with better accuracy in different experimental conditions. Specifically, Alpha1 power was positively associated with accuracy only in the presence of distractors, while Alpha2 power was negatively associated with accuracy only in the absence of distractors. We hypothesize that behavioral performance in distractor and no-distractor conditions depends on different factors. On the one hand, incorrect responses in the distractor condition might mostly be because of the interference of visual distractors. In this scenario, Alpha1 power increases become predictive of behavior because it inhibits areas involved in visual processing to avoid interference during memory retention. On the other hand, in the condition without distractors, incorrect responses might predominantly be caused by lapses of attention because of mind wandering and/or drowsiness (Braboszcz and Delorme, 2011; Andrillon et al., 2019, 2021; Rodriguez-Larios and Alaerts, 2021). Previous literature suggests that lapses of attention involve reduced cortical processing of external events (Smallwood et al., 2008). If an external event is not properly processed by the brain during an attentional lapse, its content cannot be maintained in working memory. Hence, we can expect that the recruitment of cortical areas supporting short-term memory retention (i.e., Alpha2 desynchronization) is less pronounced (or even absent) during an attentional lapse because there is little or no information to be retained. Nonetheless, it is important to note that the reported differences in accuracy depending on Alpha1/Alpha2 power modulations must be interpreted with caution because of the small number of incorrect trials (mean accuracy was 87% across conditions). In order to overcome this limitation in future work, it would be important to assess the here reported effects with a more difficult task or by adjusting task-difficulty at an interindividual level.

Recent literature points to the physiological and computational relevance of frequency modulations of neural oscillations (Cohen, 2014; Lowet et al., 2017; Klimesch, 2018). However, because of volume conduction, spurious frequency modulations at sensor level can emerge as a result of differential power modulation of multiple brain rhythms with distinct peak frequencies (Donoghue et al., 2022; Schaworonkow and Nikulin, 2022). Using ICA, we here demonstrate the existence of two distinct alpha rhythms in posterior cortex with different peak frequencies and opposite power modulations during memory retention. In light of these results, we cannot rule out the possibility that previously reported frequency changes in posterior alpha during different cognitive tasks (Angelakis et al., 2004; Haegens et al., 2014; Babu Henry Samuel et al., 2018; Rodriguez-Larios and Alaerts, 2019) are actually reflecting differential power modulations of multiple alpha rhythms with different peak frequencies. Therefore, future studies are needed to assess whether the previously reported alpha frequency changes in M/EEG during different cognitive tasks hold after the spatial separation of different alpha rhythms.

The spatial separation of different alpha rhythms could resolve some of the previous inconsistencies in the literature concerning the role of alpha phase, power and frequency in cognition (Samaha et al., 2020; Michail et al., 2022; Pavlov and Kotchoubey, 2022; Zazio et al., 2022). Similarly, separating different alpha rhythms could allow us to understand why some neurofeedback and neurostimulation protocols do not have the expected effect in some subjects (Orendáčová and Kvašňák, 2021). If we tune the neurofeedback/stimulation parameters (and/or assess their effects) at sensor level, we cannot know whether we are modulating the power or frequency of one or several alpha rhythms. As different alpha rhythms could be more prominent in different subjects because of interindividual differences in brain anatomy and functional specialization, this might have a key impact on the effects of their modulation (Duffau, 2017; Mikkonen et al., 2020).

In conclusion, our results show that posterior alpha dynamics during memory retention reflect the activity of at least two brain rhythms with distinct functions and spatiospectral characteristics. Alpha1 rhythms increased in power during memory retention and in the presence of visual distractors, while Alpha2 rhythms showed the opposite power modulations. In addition, Alpha1 and Alpha2 rhythms had an opposite relationship with accuracy (positive for Alpha1 and negative for Alpha2). Lastly, Alpha1 and Alpha2 differed significantly in several spectral parameters (peak frequency, peak width and relative amplitude) and in the location of their estimated main source. In the light of previous results and theoretical accounts (Klimesch et al., 2007; Jensen and Mazaheri, 2010; Haegens et al., 2022; Iemi et al., 2022), we hypothesize that during memory retention, Alpha1 rhythms increase in power to inhibit visual processing while Alpha2 rhythms decrease in power to disinhibit areas supporting the short-term storage of visual information.

## Synthesis

Reviewing Editor: Niko Busch, Westfalische Wilhelms-Universitat Munster Decisions are customarily a result of the Reviewing Editor and the peer reviewers coming together and discussing their recommendations until a consensus is reached. When revisions are invited, a fact-based synthesis statement explaining their decision and outlining what is needed to prepare a revision will be listed below. The following reviewer(s) agreed to reveal their identity: Christopher Benwell.

Both reviews are quite positive about the manuscript. Most of their points ask for additional clarifications or elaboration, or for straightforward control analyses. In my mind, one major concern is related to reviewer 2’s point #2: isn’t the physical difference between the distractor/no-distractor conditions a substantial confound because the physical distractor stimuli themselves are expected to induce stronger alpha desynchronization? I would ask you to pay particular attention to this point. Finally, I would like to ask you to consider making data and code publicly available.

# Reviewer 1

The authors employed MEG recordings in 33 participants to investigate the role of distinct posterior alpha components in working memory retention. Analysis of independent alpha components within participants revealed at least two dissociable alpha sources, one which increased in power during memory retention and another which decreased in power. The two alpha sources also showed differential relationships with the presence of visual distractors and opposite relationships with performance accuracy. The authors propose that the results may partially explain previous inconsistencies in the literature such as previous studies reporting both increases and decreases in alpha power during visual working memory retention.

The research question is very timely and interesting, and the study appears to be skilfully conducted using cutting edge methodologies. The manuscript is also very well written. The results provide further evidence that multiple distinct alpha components exist within participants with potentially different functional roles, and specifically link these components to visual working memory retention. The study is likely to make an important contribution to the literature. I only have a minor issue which it would be helpful if the authors could address/clarify, which I have listed below:

1. Regarding the analysis of independent components presented in Figure 3, the mean number of components significantly modulated during the memory delay across participants is provided (and SD), but I think it would be helpful if the authors could provide a more comprehensive breakdown of the distribution of significant components (both for alpha 1 and alpha 2) across participants. I think this will be helpful for interpretation, particularly of whether the two components tend to co-exist within the same participants, or rather whether individuals tend to only show either alpha 1 or alpha 2 components. I note that this information can be partially gained from the colour coding of the dots in Figure 3a, but I think it would be helpful for the reader if this information was presented explicitly in the text. For instance, how many participants showed both significant alpha 1 and alpha 2 components? How many participants only showed either significant alpha 1 or alpha 2 components? How many participants (if any) did not show any significant components?

# Reviewer 2

The behavior of alpha oscillations during visual working memory retention is debated. Mixed reports continue to appear in the literature. The authors examined this problem by decomposing MEG into alpha frequency ICA components and divided these components into Alpha1 and Alpha2 based on how they modulated by memory. They found that Alpha1 and Alpha2 are differently associated with behavior depending whether there are distractors or not during memory delay. A nice result is that they localized Alpha1 to early visual cortex and Alpha2 to higher-level visual cortex. Some comments follow.

1. I am skeptical about using ICA analysis to identify “independent” alpha components. There is no way the 170 alpha ICA components are all statistically independent. What happens if the authors bypass the ICA step and directly localize MEG signals to different part of the brain?

2. I am worried about comparing alpha power and frequency between distractor-absent memory delay and distractor-present memory delay. Distractors are visual stimuli. As such they impact alpha oscillations. One need to think of a way to account for the stimulus related alpha modulation.

3. The authors talk about upper alpha and lower alpha. Alpha1 and Alpha2 are however identified via their functional characteristic and their neural origin. Are they in any way related to upper alpha and lower alpha?

4. Alpha frequency modulation by working memory is interesting. Here is a paper that explicitly considered this problem: Babu Henry Samuel, I., Wang, C., Hu, Z., Ding, M. (2018). The frequency of alpha oscillations: Task-dependent modulation and its functional significance. NeuroImage 183, 897-906. It might help with interpreting the results.

5. “Participants reported normal vision.” All 35 have normal vision or corrected-to-normal vision?

6. It is interesting that RT is not affected by having distractors in the memory retention period. Why is that?

7. In the Results section, sensor level analysis should be marked very clearly to help the reader follow along.

8. From Figure 2B, the alpha peak became sharper during working memory retention. Any thought on what that means?

## Author Response

Synthesis Statement for Author

Both reviews are quite positive about the manuscript. Most of their points ask for additional clarifications or elaboration, or for straightforward control analyses. In my mind, one major concern is related to reviewer 2’s point #2: isn’t the physical difference between the distractor/no-distractor conditions a substantial confound because the physical distractor stimuli themselves are expected to induce stronger alpha desynchronization? I would ask you to pay particular attention to this point. Finally, I would like to ask you to consider making data and code publicly available.

Answer to Synthesis Statement

We would like to thank the editor and the reviewers for their time and their constructive feedback. We believe that their comments have helped us to improve our manuscript significantly.

We clarified several points in the text regarding the rationale, analytical approach, results and relevance of our study. In the Introduction, we elaborated on why ICA is an adequate approach for our research question. In the Methods section, we gave a more comprehensive breakdown of the alpha components. In the Results, we added a figure (Figure 3E) to illustrate that the distractor effect in alpha power is not driven by the physical difference between distractor and no distractor conditions. Finally, we clarified in the Discussion how our results could affect the interpretation of previous findings. Please see the answers to each of the comments below (changes in the manuscript’s text are marked in blue).

We plan to make data and code available on the OSF platform after publication. The link is now added in the manuscript. In this regard, we would like to ask the editor and the reviewers for their permission to also make this anonymized review publicly available on the same platform.

Reviewer #1

The authors employed MEG recordings in 33 participants to investigate the role of distinct posterior alpha components in working memory retention. Analysis of independent alpha components within participants revealed at least two dissociable alpha sources, one which increased in power during memory retention and another which decreased in power. The two alpha sources also showed differential relationships with the presence of visual distractors and opposite relationships with performance accuracy. The authors propose that the results may partially explain previous inconsistencies in the literature such as previous studies reporting both increases and decreases in alpha power during visual working memory retention.

The research question is very timely and interesting, and the study appears to be skillfully conducted using cutting edge methodologies. The manuscript is also very well written. The results provide further evidence that multiple distinct alpha components exist within participants with potentially different functional roles, and specifically link these components to visual working memory retention. The study is likely to make an important contribution to the literature. I only have a minor issue which it would be helpful if the authors could address/clarify, which I have listed below:

Comment #1

Regarding the analysis of independent components presented in Figure 3, the mean number of components significantly modulated during the memory delay across participants is provided (and SD), but I think it would be helpful if the authors could provide a more comprehensive breakdown of the distribution of significant components (both for alpha 1 and alpha 2) across participants. I think this will be helpful for interpretation, particularly of whether the two components tend to co-exist within the same participants, or rather whether individuals tend to only show either alpha 1 or alpha 2 components. I note that this information can be partially gained from the colour coding of the dots in Figure 3a, but I think it would be helpful for the reader if this information was presented explicitly in the text. For instance, how many participants showed both significant alpha 1 and alpha 2 components? How many participants only showed either significant alpha 1 or alpha 2 components? How many participants (if any) did not show any significant components?

Answer #1

We agree that explicitly mentioning the number of subjects showing Alpha1 and/or Alpha2 components would facilitate the interpretation of the results. We now added this information in the text (page 13):

Specifically, the power of 68 alpha components showed a significant increase during memory delay relative to fixation (hereafter called Alpha1), while the power of 43 alpha components showed a significant decrease (hereafter called Alpha2) (Figure 3A). The breakdown across subjects was as follows: 9 subjects showed both types of alpha components, 11 subjects showed Alpha1 components only, 7 subjects showed Alpha2 components only and 6 subjects showed no significant alpha components.

Reviewer #2

The behavior of alpha oscillations during visual working memory retention is debated. Mixed reports continue to appear in the literature. The authors examined this problem by decomposing MEG into alpha frequency ICA components and divided these components into Alpha1 and Alpha2 based on how they modulated by memory. They found that Alpha1 and Alpha2 are differently associated with behavior depending whether there are distractors or not during memory delay. A nice result is that they localized Alpha1 to early visual cortex and Alpha2 to higher-level visual cortex. Some comments follow.

Comment #1

I am skeptical about using ICA analysis to identify “independent” alpha components. There is no way the 170 alpha ICA components are all statistically independent. What happens if the authors bypass the ICA step and directly localize MEG signals to different part of the brain?

Answer #1

We apologize if our description of the results was confusing. We do not find 170 alpha components that are statistically independent per subject. Instead, we find 170 alpha components across subjects. We now clarify this in the results section (see below). Note that we also added the number of subjects that showed Alpha1 and/or Alpha2 components (see Answer #1 to Reviewer #1).

Page 13:

We found a total of 170 posterior alpha components (across subjects) based on their topography at sensor level, their spectrum, and their estimated source.

Albeit interesting, other types of source localization or source separation are beyond the scope of this manuscript. The goal of this study was to assess whether different posterior alpha rhythms are present during memory retention (as this could explain previous inconsistencies in the literature). We think that ICA is especially suitable for this purpose because it ‘simplifies’ the M/EEG inverse problem by estimating the time series of independent sources in a data-driven manner. We now elaborate on this point in the introduction (pages 4-5):

A possible explanation for the inconsistent findings regarding posterior alpha modulations during visual working memory is the existence of multiple alpha rhythms. More than 20 years ago, Klimesch et al. proposed a division of the alpha band in different sub-bands (i.e. ‘upper alpha’ and ‘lower alpha’) based on their differential power modulations during a wide range of cognitive tasks (Klimesch, 1999; Klimesch, Schimke, & Pfurtscheller, 1993). However, since alpha rhythms were not spatially separated in these studies, it was not possible to assess whether power modulations in the two alpha sub-bands reflected the activity of two different oscillators or a change in frequency of a single oscillator (Haegens, Cousijn, Wallis, Harrison, & Nobre, 2014; Mierau, Klimesch, & Lefebvre, 2017). Later studies that did perform source separation supported the idea of multiple posterior alpha rhythms. Specifically, spatially distinct posterior alpha rhythms have been identified in the context of resting state (Barzegaran, Vildavski, & Knyazeva, 2017), visual perception (Benwell et al., 2019; Gulbinaite, İlhan, & Vanrullen, 2017) and multimodal attention (Sokoliuk et al., 2019).

Previous literature has shown that independent component analysis (ICA) is a powerful analytical tool to separate brain rhythms in M/EEG (Benwell et al., 2019; Debener et al., 2005; Wagner, Wessel, Ghahremani, & Aron, 2018). ICA is a blind source separation technique that aims to identify signals whose time courses convey maximally distinct information. When applied to M/EEG data, ICA is thought to be able to separate locally synchronous activity from distinct ‘cortical patches’ (i.e. compact neuronal populations with strong short-range anatomical connections) (Onton, Westerfield, Townsend, & Makeig, 2006). The main advantage of ICA relative to other source localization techniques is that it simplifies the M/EEG inverse problem by modelling which signals are contained in the volume conducted scalp data instead of estimating the projection weights of all possible sources (Delorme et al., 2012).

Comment #2

I am worried about comparing alpha power and frequency between distractor-absent memory delay and distractor-present memory delay. Distractors are visual stimuli. As such they impact alpha oscillations. One need to think of a way to account for the stimulus related alpha modulation.

Answer #2

We thank the reviewer for raising this relevant point. Previous literature has indeed shown that visual stimuli can disrupt ongoing alpha oscillations by the generation of an event-related potential (Mazaheri & Jensen, 2006). If alpha power modulations in the distractor condition would be due to event-related responses to visual distractors, we would expect alpha power to decrease in the presence of distractors. However, our results show that the presentation of visual distractors during the memory delay affects the two alpha rhythms differently (Alpha1 increases in power while Alpha2 decreases). Hence, the presentation of visual distractors modulates but does not change the direction of alpha power dynamics during the memory delay. This rules out the possibility that distractor-related alpha power modulations are simply due to the physical properties of the visual stimuli presented during the memory delay.

We now provide a better visualization of the distractor effect in Figure 3E to clarify this point (see below). Specifically, we now plot the grand average time-frequency representations of Alpha1 and Alpha2 components for the distractor and the no-distractor conditions. In these plots we can clearly observe that distractors modulate-but do not change the direction of- Alpha1/Alpha2 power dynamics during the memory delay.

Comment #3

The authors talk about upper alpha and lower alpha. Alpha1 and Alpha2 are however identified via their functional characteristic and their neural origin. Are they in any way related to upper alpha and lower alpha?

Answer #3

This is a great question. Our results are difficult to compare with previous studies that distinguished two sub-bands in the alpha range (i.e. ‘upper alpha’ and ’lower alpha’) (Klimesch, 1999). In our study, we find two alpha rhythms with different functions and spatiospectral characteristics in occipito-parietal cortex. Instead, the original division in upper and lower alpha was based on the average EEG spectrum (Doppelmayr, Klimesch, Pachinger, & Ripper, 1998). Crucially, it has been shown that alpha oscillations are not limited to posterior areas (Iemi et al., 2022). Some examples of non-posterior alpha rhythms are the auditory ’tau’ rhythm and the sensorimotor ’mu’ rhythm (Bastarrika-Iriarte & Caballero-Gaudes, 2019; Haegens, Händel, & Jensen, 2011; Lehtelä, Salmelin, & Hari, 1997; Pfurtscheller, Neuper, & Krausz, 2000). Consequently, it is likely that the upper and lower alpha bands as previously described contain a mix of more than two rhythms (including Alpha1 and Alpha2).

Comment #4

Alpha frequency modulation by working memory is interesting. Here is a paper that explicitly considered this problem: Babu Henry Samuel, I., Wang, C., Hu, Z., Ding, M. (2018). The frequency of alpha oscillations: Task-dependent modulation and its functional significance. NeuroImage 183, 897-906. It might help with interpreting the results.

Answer #4

We agree that frequency modulation during working memory is of functional relevance. In fact, this is the reason we believe it is important to first isolate the rhythm of interest. Otherwise, frequency changes can be easily confounded by power changes of several rhythms with different peak frequencies. We now clarify this point in the discussion (page 21):

Recent literature points to the physiological and computational relevance of frequency modulations of neural oscillations (Cohen, 2014; Klimesch, 2018; Lowet, Roberts, Peter, Gips, & De Weerd, 2017). However, due to volume conduction, spurious frequency modulation at sensor level can emerge as a result of differential power modulation of multiple brain rhythms with distinct peak frequencies (Donoghue, Schaworonkow, & Voytek, 2021; Schaworonkow & Nikulin, 2022a). Using ICA, we here demonstrate the existence of two distinct alpha rhythms in posterior cortex with different peak frequencies and opposite power modulations during memory retention. In light of these results, we cannot rule out the possibility that previously reported frequency changes in posterior alpha during different cognitive tasks (Angelakis, Lubar, Stathopoulou, & Kounios, 2004; Babu Henry Samuel, Wang, Hu, & Ding, 2018; Haegens et al., 2014; Rodriguez-Larios & Alaerts, 2019) are actually reflecting differential power modulations of multiple alpha rhythms with different peak frequencies. Therefore, future studies are needed to assess whether the previously reported alpha frequency changes in M/EEG during different cognitive tasks hold after the spatial separation of different alpha rhythms.

Comment #5

"Participants reported normal vision.” All 35 have normal vision or corrected-to-normal vision?

Answer #5

Participants had normal or corrected-to-normal vision. We added this information in the methods section (page 6):

Participants reported normal or corrected-to-normal vision and no history of neurological or mental illnesses.

Comment #6

It is interesting that RT is not affected by having distractors in the memory retention period. Why is that?

Answer #6

Since we use response mappings (i.e. decision-button association changes in each trial), reaction times are difficult to interpret in this task. This is why we focused on accuracy for brain-behavior associations. We now add this information in the results section (page 12):

No effect of distractor nor rule by distractor interaction was found. Note that since the decision-button association changed in each trial, reaction times are difficult to interpret in this task. For this reason, we focused on accuracy to assess brain-behavior associations.

Comment #7

In the Results section, sensor level analysis should be marked very clearly to help the reader follow along.

Answer #7

We now changed the name of the respective section to: Posterior alpha peak at sensor-level increases in power and decreases in frequency during memory retention.

Comment #8

From Figure 2B, the alpha peak became sharper during working memory retention. Any thought on what that means?

Answer #8

It is difficult to interpret spectral changes at M/EEG sensor level because they reflect a spatial mix of different brain sources (Schaworonkow & Nikulin, 2022b). Based on our ICA results, we can speculate that the peak of sensor-level alpha becomes narrower during memory retention because the Alpha1 rhythm becomes more prominent relative to the Alpha2 rhythm. As depicted in Figure 4A, Alpha1 rhythms have a sharper profile than Alpha2 rhythms (see below).
